# Hypertrophy of the Prostrate Gland

**Published:** 1919-01

**Authors:** Gustav Kolischer

**Affiliations:** Chicago, Ill.; Attending Surgeon to the G. U. and Radiotherapeutic Department of Michael Reese Hospital, Professor of G. U. Surgery, Chicago Post-Graduate School, Chicago


### ORIGINAL ARTICLES.

### Hypertrophy of the Prostate Gland.

G. KOLISCHER, M. D., CHICAGO, ILL.

Attending Surgeon to the G. U. and Radiotherapeutic Department of Michael Reese Hospital, Professor of G. U. Surgery, Chicago Post-Graduate School, Chicago.

Hypertrophy of the prostate gland is an idiopathic tumefaction peculiar to the advanced age. While all kinds of theories were advanced in an effort to explain its origin, none of them proved to be satisfactory. In a general way we may characterize the prostatic hypertrophy as a symmetric and asymmetric one, according to the preponderance of the enlargement in only one sector of the gland or extending uniformly all over the organ.

Modern investigations have demonstrated that hypertrophy always concerns the central parts of the gland, and that the so-called surgical capsule of the prostate is also prostatic tissue, the peripheral portion of the gland being compressed by and separated to a greater or lesser extent from the central glandular mass. In a histologic sense the prostatic enlargement presents itself either as hard hypertrophy, the fibro-muscular or myomatous form, or as soft hypertrophy, the glandular or adenomatous type. Both these types may occur in the same specimen.

Of great practical importance are the changes befalling the urethra of the prostate, which changes are apt to influence the length, the width and the course of the urethra.

The posterior urethra becomes always elongated during the growing of the prostatic intumescence; to its normal length of about three centimeters, two to four centimeters may be added according to the size of the prostatic tumor, that is tunneled by the posterior part of the urethra.

Along with the protrusion of the prostate into the bladder, the internal urethral orifice is carried along and as a rule is elevated into the cavity of the viscus.

The diameter of the prostatic urethra is always enlarged, sometimes to such an extent that the prostatic part of the urethra is transformed into a sac, that permits rotary movements of a shortbeaked instrument. This condition may lead to the erroneous conception that the beak of the introduced catheter or sound has already penetrated into the bladder, although it still is inside the urethra. The normal direction of the urethra is mainly interfered with in cases of asymmetrical hypertrophy, which interference in extreme cases may lead to kinking of the urethra, thus making the introduction of an instrument very difficult or even impossible.

The clinical dignity of prostatic hypertrophy is determined mainly by the degree of impediment offered to the prompt and complete evacuation of the urinary bladder.

If a valvular or pedunculated lobe lies against the internal urethral orifice, the urination may be started very slowly and occasionally be interrupted, but as a rule in certain positions, especially if preceded by movements of the trunk, the passage is set free again and the emptying of the bladder may be completed.

The situation is very different if the hypertrophy is of such a character as to change the normal topography of the internal meatus. The internal urethral orifice is normally the lowest point of the bladder. Whenever this position of the urethral entrance is changed, urinary difficulties will arise, because this part of the bladder that is on a lower level than the urethral mouth cannot be emptied through it by the contraction of the detrusor muscle. Consequently a certain amount of urine will always remain in the bladder after each micturition, which remnant is called the residual urine.

The quantity of this residual urine is in direct proportion to the size of the pouch that is formed underneath the internal urethral orifice, or, in other words, proportional to the degree of the elevation of the internal meatus into the cavity of the bladder.

The presence of residual urine is ascertained by catheterizing the patient immediately after he finishes an act of spontaneous urination, while the measuring of the quantity of urine drawn by instrumentation gives information as to the amount of the residual urine.

It is evident that a large intumescence of the prostatic gland, mainly developed toward the rectum, will offer less interference with urination than a hypertrophy of lesser degree that encroaches mainly on the vesical cavity. A distinction between these two forms is essential for prognosis and indication of treatment. Direct inspection in the course of a cystoscopy of course will furnish exact information as to these details, but there are also other ways to arrive at satisfying conclusions concerning the character of the hypertrophy in a given case. Firstly, if the posterior urethra is considerably elongated, we are justified in assuming thatt he internal urethral orifice is carried far into the bladder; this excessive elongation of the prostatic urethra is revealed by the fact that an unusual length of the catheter has to be introduced before the urinary flow is started.

In lean individuals a bimanual examination undertaken immediately after catheterization will enable one to palpate the prostatic tumor and to outline its extension into the bladder. In obese patients, in which this examination will not furnish satisfactory results, a sizable protrusion of the enlarged prostate into the bladder may be diagnosed, if intermittent pressure of one hand placed on the abdomen on the vesical region succeeds in communicating an impulse to a finger placed in the rectum. Again, if pressure brought to bear on the vesical region on either side of the median line succeeds in accelerating or restarting the urinary flow through an introduced catheter, the diagnosis of a prostatic tumor protruding high up into the bladder will be made.

In case the prostatic tumor be of such a size that it practically divides the bladder in two halves a very interesting phenomenon may be demonstrated. If the urinary stream is started by the passing of a catheter, pressure on the median line over the vesical area will stop the flow, because the upper or anterior wall of the bladder is forced against the eye of the catheter, that emanates from the top of the summit produced by the excessive hypertrophy of the prostate, which protrusion is carrying the internal urethral orifice on its crown. The subjective symptoms depend on the character of the prostatic tumor, on the presence of concomitant urethral stricture, on the condition of the detrusor muscle and upon the absence or existence of local infection.

In the beginning there may be nothing else but slowness in starting the urinary flow, then frequency of urination, which becomes particularly noticeable after the patient becomes warm in bed; this frequency may grade from two or three urinary calls during the night up to well developed disturbance of the night rest.

Patients with large soft prostates are permanently conscious of their prostate, they complain about feeling a lump in the lowest segment of the rectum and also sense this lump as the seat of a permanent sensation of heat.

A rapidly growing prostate becomes the location of quite intense pains produced by the quickly increasing tension, which pains may irradiate into one or both thighs, stimulating sciatica.

This pressure may also lead to very pronounced failing of the sexual power.

While in the initial stages of hypertrophy the urinations, as a rule, will be started and finished mayhap under exertion and by calling in for help the abdominal pressure, even at this period acute and complete retention may set in if the patient, in not yielding to an urgent urinary call, permits his bladder to become overdistended. The same may occur during an alcoholic excess, when timely urination is missed, because the epithelial and muscular sensation is dulled by intoxication.

A patient being the carrier of an old stricture, that is not too tight, or is kept open by regular sounding, for a long time may be able to overcome a moderate prostatic obstruction, because his bladder has developed a labor hypertrophy, the detrusor muscles having been compelled to force the urinary stream through a partially hardened and narrowed urethra.

On the other hand, an irritable stricture under a slight provocation may add to the impediment of the urinary flow by causing the establishing of a retrostrictural edema, causing a temporary swelling of the prostate to such a degree that complete retention of urine results.

In line with the development of chronic partial retention and increase of the amount of residual urine the patients begin to complain about the lack of satisfaction after each urination, the backpressure leading to pains in the ureteral and renal regions. In extreme cases the patients lose constantly small quantities of urine, the result of a permanent overflowing of the bladder, which phenomenon is known as paradoxical incontinence, a permanent dribbling of urine, although the bladder remains filled all the time.

The establishing of infection in the bladder, invariably occurring in prostatic hypertrophy of long standing, leads to an exacerbation of all the symptoms.

The bladder and prostate are the seat of a permanent ache with occasional exacerbations, each urination is painful and followed by dolorous spasms, which in extreme cases may lead to regular deliria of the detrusor and the perineal muscles.

Loss of sleep and the intoxication due to the absorption of the toxins of the inflammatory products emaciate the patient, nightly fever is nothing unusual. Invasion of the kidneys is marked by chills, pains in the renal regions, and palpatory sensitiveness in one or both kidneys.

The urine becomes pussy, acquires an offensive odor and is very thick. Regular catheterization will furnish relief, almost complete in the initial stages, and somewhat helpful in later periods for two reasons. The bladder is regularly and completely emptied, and antiseptic irrigations following the evacuation may prevent for quite a while infection and inflammation, and are apt to improve somewhat on the symptoms due to an already established infection and its inflammatory sequelae.

Catheterization has always to be guarded by the strictest aseptic precautions. In case of repeated and complete retention and overdistension of the bladder the bladder should not be emptied at once, in order to avoid a hemorrhage, as vacuo and too sudden changing of the intrarenal tension by suddenly and completely relieving the backpressure. The emptying of the bladder has to be completed at easy stages. The empty bladder has to be flushed by an antiseptic solution, a small quantity of which is left in the bladder, to interfere as much as possible with the proliferation of the pathogenic germs that have invaded the bladder.

The catheterization should always be attempted firstly with a soft rubber catheter of as large a caliber as the external meatus will admit; failure to succeed will entail the application of an elastic (Mercier) catheter. The instrumentation will be facilitated and made possible even in very difficult instances, if previously to the introduction of a catheter a few c. c. of the lubricant used is injected into the urethra. Only if even this latter attempt should fail, the use of a silver catheter may be resorted to; such an instrument has to be used with the utmost caution; the operator should never permit himself to use any appreciable force, in order to avoid the boring of a false route in the urethra or a perforation of the prostate, both accidents occurring much easier than is generally believed.

Absolute impossibility of catheterization will call for the emptying of the bladder through a suprapubic puncture.

This is at best done by the use of a fine aspirating needle, and through this the bladder is pumped out. The puncture of the bladder with a troicar of a large calibre is better avoided. Quite often the peritoneum is drawn over the bladder and adherent to the symphysis and it will remain in this topographic position, although the bladder may be distended ad maximum.

As large an opening as produced by a troicar is dangerous on account of the initial size of the perforation and on account of the subsequent leakage of the urine, not to speak of the danger of a large puncture penetrating into a bowel.

